# Frailty and prolonged preoperative waiting time as independent predictors of postoperative delirium in older hip fracture patients

**DOI:** 10.1038/s41598-025-32904-z

**Published:** 2025-12-23

**Authors:** Yuzhi Wei, Haotian Wu, Chunyu Feng, Yujie Wang, Ziheng Qi, Huan Zhang

**Affiliations:** 1 School of Clinical Medicine, Tsinghua University, Beijing, China; 2 Department of Anesthesiology, Beijing Tsinghua Changgung Hospital, No.168 Litang Road, Changping District, Beijing, 102218 China; 3 Medical Data Science Center, Beijing Tsinghua Changgung Hospital, No.168 Litang Road, Changping District, Beijing, 102218 China

**Keywords:** Postoperative delirium, Hip fracture, Frailty, Preoperative waiting time, Postoperative infection, Diseases, Health care, Medical research, Risk factors

## Abstract

Postoperative Delirium (POD) is a relatively common acute neurocognitive complication in elderly patients with hip fractures. However, its incidence and independent risk factors remain incompletely defined. This analysis utilized prospectively collected data from 238 consecutive patients with hip fractures aged ≥ 60 years. Univariate analyses were first used to assess potential risk factors for POD. Variables showing significant associations underwent collinearity testing, and those with high variance inflation factors (VIF > 5.0) were excluded. To ensure model stability per the Events Per Variable (EPV) principle and to focus on clinically meaningful predictors, a final set of 7 variables was entered into a multivariate logistic regression model to identify independent predictors of POD. The analysis included 238 patients with an average age of 79.0 years, of whom 73.1% were women. The overall incidence of POD was 31.9%. Those who developed POD were significantly older (82.5 vs. 77.3 years) and a higher proportion were female (77.6% vs. 71.0%). Multivariate analysis identified four independent predictors of POD: frailty (OR = 2.62, 95% CI 1.22–5.62, *P* = 0.014), a preoperative waiting time exceeding 90 h (OR = 2.59, 95% CI 1.31–5.15, *P* = 0.007), postoperative infection (OR = 2.41, 95% CI 1.21–4.82, *P* = 0.013), and advanced age over 82 years (OR = 2.44, 95% CI 1.26–4.73, *P* = 0.008). Patients with POD experienced longer ICU stays (median 17.8 days vs. 0 days, *P* < 0.001) and longer total hospitalization (13 days vs. 10 days, *P* < 0.001). Frailty, prolonged preoperative waiting time, postoperative infection, and advanced age were identified as independent predictors of POD in older hip fracture patients. These findings may help improve understanding of delirium risk in this population and suggest areas for further investigation. Further studies are warranted to explore causal mechanisms and to evaluate whether perioperative interventions aimed at reducing waiting time, assessing frailty, or preventing infections might mitigate the risk of POD.

## Introduction

 Hip fracture remains a major clinical challenge in the aging population, representing a leading cause of functional decline and increased long-term care dependency. Against this backdrop, POD has emerged as a frequent and serious neuropsychiatric complication in older hip fracture patients, with reported incidence rates ranging from 5 to 61%^1–3^, is recognized as a serious complication that independently predicts poor long-term functional outcomes^4–6^. Clinically, it is categorized into hyperactive, hypoactive, and mixed subtypes; however, it remains substantially underdiagnosed—only 20.9% of cases are systematically documented, and up to 75% are overlooked in emergency settings^7^, underscoring critical gaps in recognition and diagnosis.

The rising prevalence of hip fractures amid global aging further compounds this issue, particularly given the vulnerability of this patient cohort. These individuals often present with osteoporosis (prevalent in 69% of fragility fracture cohorts^8^, impaired ambulation, and complex multimorbidity profiles^8,9^, which may increase susceptibility to POD. This complication exerts considerable bidirectional strain on both the healthcare system and patient outcomes. Evidence indicates that POD is associated with a 36% prolongation of ICU stay and a 22% increase in total hospitalization duration^10^. Moreover, it extends rehabilitation time by 6.5 days, elevates the risk of pressure ulcer^11,12^ and surgical site infections (OR = 4.38)^13^, and is significantly associated with increased long-term mortality (OR = 2.11)^14^.

Previous systematic reviews and meta-analyses have consistently identified several core risk factors for POD in this population, such as advanced age, preoperative cognitive impairment, and specific comorbidities^15,16^. While acknowledging these foundations, such studies have concurrently highlighted knowledge gaps regarding additional modifiable factors like frailty and prolonged waiting time. Among these, frailty is of particular interest due to its multifaceted mechanistic links to POD. Frailty, defined by diminished physiological reserve and impaired stress response, predisposes patients to POD through interconnected mechanisms. Chronic inflammation, often termed “inflammatory aging,” marked by elevated pro-inflammatory cytokines such as interleukin-6 and tumor necrosis factor-α, disrupts the integrity of the blood-brain barrier^17,18^. This inflammatory burden is compounded by mitochondrial dysfunction^19^, which generates oxidative stress, triggers neuronal apoptosis, and impairs synaptic plasticity. Additionally, hypothalamic-pituitary-adrenal (HPA) axis dysregulation disrupts cortisol rhythm, contributing to cognitive fluctuations^20^. These insights highlight the potential of targeted frailty interventions as innovative strategies to mitigate the risk of POD^21^.

Therefore, this study aimed to address these specific gaps by identifying key risk factors using a substantial prospectively collected dataset, while also evaluating POD’s impact of POD on critical outcomes, including ICU stay, hospitalization duration, and postoperative complications. Our findings may inform targeted preventive strategies to improve care and reduce the associated healthcare burden.

## Materials and methods

### Study design

This was a prospective cohort study; all data collection for exposures and outcomes occurred after study initiation and followed patients forward in time.

This prospective cohort study was conducted at Beijing Tsinghua Chang Gung Hospital. It included patients aged ≥ 60 years with imaging-confirmed hip fractures requiring surgery. Patients were consecutively enrolled between January 2022 and November 2023. The study protocol was approved by the institutional ethics committee (No. 21277-0-01), and all methods were performed in accordance with the relevant guidelines and regulations. The trial was prospectively registered at ClinicalTrials.gov (NCT05246254). All participants provided witnessed written informed consent after trained researchers explained the study procedures. Data collection used standardized case report forms integrated with an electronic medical record system for real-time perioperative data verification.

### Inclusion and exclusion criteria

This prospective cohort study enrolled 238 patients hospitalized for hip fractures between January 1, 2022, and November 30, 2023. The inclusion criteria were as follows: age ≥ 60 years, presence of hip fracture, signed informed consent form, American Society of Anesthesiologists (ASA) class I-IV, and surgery performed by a consistent anesthetic and surgical team. Exclusion criteria included: inability to provide informed consent; conservative treatment without surgery; duplicate fracture records (referring to instances where the same fracture episode was inadvertently entered into the screening log more than once); inability to complete cognitive function tests; delirium during initial assessment; diagnosed psychiatric or substance use disorders; and incomplete or missing follow-up data. All patients received standardized preoperative preparation, anesthesia, and postoperative management. The primary objective of this study was to evaluate the occurrence of POD.

### Standardized anesthetic management protocol

A standardized anesthetic protocol was implemented for all patients. The operating room environment was maintained at 20–23 °C with 50–60% humidity. Active warming was achieved using forced-air blankets and fluid warmers to sustain normothermia (36.5–37.2 °C).

All patients received continuous monitoring including electrocardiography, non-invasive blood pressure, and pulse oximetry. For neuraxial anesthesia, combined spinal-epidural anesthesia was performed at L2-3/L3-4 interspaces using 0.75% isobaric ropivacaine (1.8–2.2 mL), followed by epidural catheter placement. Sedation was maintained with dexmedetomidine infusion (0.1–0.3 µg/kg/h) targeting Ramsay Sedation Scale scores of 2–3.

For patients requiring general anesthesia, induction included etomidate or propofol with sufentanil and rocuronium. Anesthesia was maintained with propofol or dexmedetomidine infusion combined with remifentanil and sevoflurane, targeting bispectral index values of 40–60. Invasive arterial monitoring was utilized for ASA class ≥ III patients or those with significant cardiopulmonary comorbidities. All patients received multimodal analgesia including ultrasound-guided continuous femoral nerve block.

### Observation indicators

Preoperative variables included age, sex, BMI, smoking history, alcohol abuse, comorbidities (hypertension, heart disease, diabetes mellitus, kidney diseases, history of stroke), and Charlson Comorbidity Index (CCI), with patients categorized into low (≤ 2) and high (> 2) comorbidity burden groups. Nutritional status was assessed using the Mini Nutritional Assessment (MNA) scale, classifying participants as malnourished (< 17), at risk of malnutrition (17–23.5), or with a normal nutritional status (≥ 24). Frailty status, based on the Frailty Index (FI)^22^, divided participants into non-frail (FI < 0.25) and frail (FI ≥ 0.25) status. Other preoperative factors included medication history, fracture type and side, presence of multiple fractures, history of contralateral hip fracture, preoperative laboratory test results, Prognostic Nutrition Index (PNI), preoperative waiting time (from admission to surgery), and ASA classification. Operative variables comprised surgical method, anesthesia type, operation duration, and estimated blood loss. Postoperative variables covered complications (venous thromboembolism, urinary tract infections, pulmonary infections, myocardial infarction, stroke, gastrointestinal hemorrhage), hemoglobin level on postoperative day 1, ICU stay duration, and total hospital length of stay.

### Outcome measures

The Confusion Assessment Method (CAM)^23,24^, the internationally validated gold-standard instrument for delirium detection, was systematically implemented with its four diagnostic pillars: (1) acute onset/fluctuating course, (2) inattention, (3) disorganized thinking, and (4) altered consciousness. Demonstrating exceptional psychometric properties (sensitivity: 95–100%; specificity: 90–95%), this protocol enabled standardized identification of both hyperactive and hypoactive delirium subtypes. Certified delirium assessments were conducted by a trained research team, which included clinical research nurses performing twice-daily evaluations under the supervision of a consultant geriatrician specialized in cognitive disorders. This multi-rater, instrument-based approach was implemented to ensure consistent and objective diagnosis. Assessments were carried out at critical postoperative intervals (POD 1, 2, 3, 5, 7) using time-series CAM evaluations to capture dynamic symptom trajectories. The rigorous assessment framework incorporated serial neurological examinations to differentiate transient cognitive changes from true delirium episodes.

### Outcome indicators

The primary outcome was the occurrence of POD, which was diagnosed using the Confusion Assessment Method (CAM). Secondary outcomes included the hospital length of stay and the ICU length of stay (defined as the duration from ICU admission to transfer to a general ward). Outcome assessors received standardized training in delirium evaluation protocols prior to data collection.

### Sample size estimation

The required sample size was estimated a priori based on data from our previous cohort study^25^. In that study, the incidence of POD was 42.3% (30/71) in frail patients versus 7.8% (6/77) in non-frail patients following hip fracture surgery. Utilizing the established proportions (p1 = 0.423, p0 = 0.078), a two-sided alpha of 0.05, and 90% power (β = 0.1), the minimum sample size necessary to detect this difference was calculated to be 118 participants (59 per group) employing the standard formula for comparing two proportions. The final cohort of 238 patients therefore provided sufficient statistical power (> 99%) for the primary analysis.

### Statistical analysis

The analytical cohort was stratified based on the occurrence of POD within 7 postoperative days. The normality of continuous variables was assessed using the Kolmogorov-Smirnov test. Data that is normally distributed (for example, metabolic and nutritional parameters) is presented as mean (SD); data that is not normally distributed (for example, surgical parameters and length of stay) is expressed as the median and interquartile range (IQR). Categorical variables are summarized as frequencies and percentages. Intergroup comparisons employed Student’s *t*-test or the Mann-Whitney U test for continuous variables, and the Chi-square or Fisher’s exact test for categorical variables, as appropriate.

Univariate logistic regression was performed to identify variables associated with POD. All variables with a significance level of *p* < 0.05 in univariate analysis were assessed for multicollinearity using VIF diagnostics. Variables with a VIF > 5 were excluded from subsequent multivariate analysis.

The observed 76 POD events were used to assess the stability of the multivariable model, with the EPV principle guiding the inclusion of approximately 7 predictors. In order to adhere to this principle and focus on core pathophysiological pathways, urinary tract and pulmonary infections were combined into a composite “postoperative infection” variable. The final multivariable logistic regression model was adjusted for the following covariates: age > 82 years, frailty status, preoperative waiting time > 90 h, postoperative infection, nutritional status (MNA scale), CCI score, and ASA physical status classification.

Adjusted odds ratios with 95% confidence intervals were computed, and all statistical analyses were performed using SPSS (version 27.0; IBM Corp., Armonk, NY, USA). A two-sided *p* value < 0.05 was considered statistically significant.

## Results

**Fig. 1:**
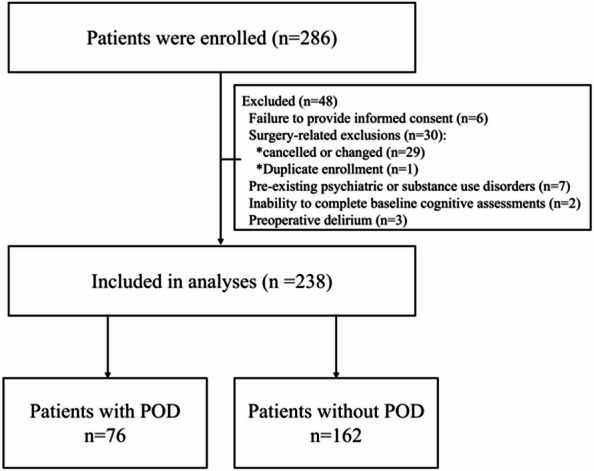
Depicts the flowchart of patient enrollment, with POD denoting postoperative delirium.

In the period spanning from January 2021 to November 2023, a total of 286 patients were systematically evaluated for their suitability for inclusion. Of these, 238 satisfied the stipulated inclusion criteria and were consequently enrolled in the present cohort (see Fig. 1 for details). The collection of all prespecified outcome data was conducted prospectively, with complete follow-up and no missing data.

### Characteristics of the study population

The study cohort comprised 238 older hip fracture patients with mean age 79.0(SD 8.7) years (females: 73.1%, *n* = 174). POD occurred in 76 cases (31.9%). Comparative analysis revealed significant intergroup disparities in demographic and clinical profiles: POD patients demonstrated significantly higher prevalence of advanced age (> 82 years: 63.2% vs. 34.0%, *p* < 0.001), preoperative frailty (76.3% vs. 34.6%, OR = 6.10, 95% CI 3.28–11.34), and malnutrition (22.4% vs. 4.3%, *p* < 0.001). Nutritional biomarkers showed marked deterioration in POD group - total albumin (66.8 [SD 5.5] vs. 69.3 [SD 5.4] g/L, *p* < 0.001) and PNI (37.2 [SD 4.0] vs. 39.5 [SD 4.0], *p* < 0.001). The mean preoperative waiting time was 65.5 h. Delays beyond 48 h were primarily due to medical optimization of comorbidities and operational constraints, reflecting real-world clinical practice at our center during the study period. Comparative analysis of anesthetic modalities revealed a predominance of combined spinal anesthesia over general anesthesia in both POD and non-POD cohorts (67.1% vs. 32.9% and 72.2% vs. 27.8%, respectively), though these proportional differences lacked statistical significance (*p* > 0.05). Similarly, no significant intergroup disparities emerged in operative strategy selection, with comparable utilization rates of arthroplasty versus internal fixation (46.6% vs. 53.4%, *p* = 0.337) across study populations, as detailed in Table 1. These findings suggest that neither anesthetic modality nor surgical approach independently contributed to delirium risk stratification in this cohort.

**Table 1:** Comparison of baseline characteristics and other covariables between patients with and without postoperative delirium (POD).

Parameter	Total n = 238	With POD n = 76	Without POD n = 162	*p* value
Age**, years	79.0 (8.7)	82.5 (7.8)	77.3 (8.6)	< 0.001
Gender, n (%)				0.281
Female	174 (73.1)	59 (77.6)	115 (71.0)	
Male	64 (26.9)	17 (22.4)	47 (29.0)	
BMI, kg/m^2^	24.0 (4.4)	23.3 (4.7)	24.3 (4.3)	0.110
Active smoking, n (%)	12 (5.0)	3 (3.9)	9 (5.6)	0.757
Alcohol abuse, n (%)	6 (2.5)	1 (1.3)	5 (3.1)	0.667
Comorbidity, n (%)				
Hypertension*	163 (68.5)	59 (77.6)	104 (64.2)	0.038
Heart disease	90 (37.8)	33 (43.4)	57 (35.2)	0.222
Diabetes mellitus	76 (31.9)	22 (28.9)	54 (33.3)	0.499
Kidney disease	15 (6.3)	5 (6.6)	10 (6.2)	1.000
Respiratory diseases*	178 (74.8)	65 (85.5)	113 (69.8)	0.009
History of stroke*	68 (28.6)	29 (38.2)	39 (24.1)	0.025
CCI**, Median (IQR)	5.0 (4.0, 6.0)	6.0 (5.0, 7.0)	4.0 (3.0, 5.0)	< 0.001
MNA scale**, n (%)				< 0.001
Normal nutrition	56 (23.5)	9 (11.8)	47 (29)	
At risk	158 (66.4)	50 (65.8)	108 (66.7)	
Malnourished	24 (10.1)	17 (22.4)	7 (4.3)	
Frailty**, n (%)	114 (47.9)	58 (76.3)	56 (34.6)	< 0.001
Medication history, n (%)				
Hormone	11 (4.6)	3 (3.9)	8 (4.9)	1.000
Zoledronic acid	5 (2.1)	0 (0)	5 (3.1)	0.180
Type of fracture, n (%)				0.286
Femoral neck	128 (54.2)	35 (47.3)	93 (57.4)	
Intertrochanteric	104 (44.1)	38 (51.4)	66 (40.7)	
Subtrochanteric	4 (1.7)	1 (1.4)	3 (1.9)	
Side, n (%)				0.779
Left	118 (50.0)	38 (51.4)	80 (49.4)	
Right	118 (50.0)	36 (48.6)	82 (50.6)	
Multiple fractures, n (%)	31 (13.0)	10 (13.2)	21 (13)	0.967
Contralateral hip fracture, n (%)	22 (9.2)	9 (11.8)	13 (8)	0.343
Preoperative laboratory test				
Serum sodium, mmol/L	138.6 (5.0)	139.0 (5.8)	138.4 (4.6)	0.353
Serum potassium, mmol/L	3.9 (0.5)	3.9 (0.5)	3.8 (0.5)	0.648
Serum calcium, mmol/L	2.2 (0.2)	2.2 (0.2)	2.2 (0.1)	0.934
Total albumin**, g/L	68.5 (5.5)	66.8 (5.5)	69.3 (5.4)	0.001
Albumin**, g/L	38.8 (4.1)	37.2 (4.0)	39.5 (4.0)	< 0.001
Absolute lymphocyte count, × 10⁹/L	1.1 (0.5)	1.1 (0.5)	1.2 (0.5)	0.437
PNI**, Median (IQR)	38.8 (4.1)	37.2 (4.0)	39.5 (4.0)	< 0.001
Preoperative waiting time**, Median (IQR), hours	65.5 (39.0, 96.0)	88.0 (47.0, 120.0)	50.5 (33.1, 86.1)	< 0.001
Preoperative waiting time > 90 h**, n (%)	71 (29.8)	37 (48.7)	34 (21)	< 0.001
ASA classification**, n (%)				< 0.001
Class I	0 (0.0)	0 (0.0)	0 (0.0)	
Class II	25 (10.5)	1 (1.3)	24 (14.8)	
Class III	191 (80.6)	62 (82.7)	129 (79.6)	
Class IV	21 (8.9)	12 (16)	9 (5.6)	
Type of anesthesia, n (%)				0.419
General anesthesia	70 (29.4)	25 (32.9)	45 (27.8)	
Spinal anesthesia	168 (70.6)	51 (67.1)	117 (72.2)	
Surgical Methods, n (%)				0.337
Arthroplasty	111 (46.6)	32 (42.1)	79 (48.8)	
Internal fixation	127 (53.4)	44 (57.9)	83 (51.2)	
Operation time, Median (IQR), hours	2.0 (1.5, 2.0)	2.0 (1.5, 2.0)	2.0 (1.5, 2.4)	0.386
Estimated blood loss, Median (IQR), mL	100.0 (50.0, 200.0)	100.0 (50.0, 200.0)	100.0 (50.0, 200.0)	0.285
Postoperative complications, n (%)				
Venous Thromboembolism	79 (33.2)	26 (34.2)	53 (32.7)	0.819
Urinary tract infections**	69 (29.0)	36 (47.4)	33 (20.4)	< 0.001
Pulmonary infections**	21 (8.8)	14 (18.4)	7 (4.3)	< 0.001
Myocardial infarction	5 (2.1)	4 (5.3)	1 (0.6)	0.037
Stroke	11 (4.6)	6 (7.9)	5 (3.1)	0.110
Gastrointestinal bleeding	2 (0.8)	1 (1.3)	1 (0.6)	0.538
Incision infection	2 (0.8)	2 (2.6)	0 (0)	0.101
Hb on Day 1 post-operation, Median (IQR), g/L	97.0 (87.0, 108.0)	94.0 (85.0, 103.8)	97.0 (88.0, 108.0)	0.127
ICU time**, Median (IQR), days	0.0 (0.0, 20.0)	17.8 (0.0, 24.2)	0.0 (0.0, 16.0)	< 0.001
Length of stay**, Median (IQR), days	10.5 (8.0, 14.0)	13.0 (9.0, 16.0)	10.0 (8.0, 12.0)	< 0.001

POD, postoperative delirium; BMI, Body mass index; CCI, Charlson comorbidity index; MNA scale, Mini nutritional assessment scale, "Nutritional status was assessed using the Mini Nutritional Assessment (MNA). Participants were classified into three groups based on MNA scores: malnourished (< 17), at risk of malnutrition (17–23.5), and normal nutritional status (≥ 24)."; PNI, Prognostic Nutritional Index; ASA, American society of anesthesiologists; ICU, intensive care unit.

*p value < 0.05 is statistically significant, **p value < 0.001 is highly significant.

Data are presented as mean (SD) unless otherwise indicated.

Patients with POD exhibited a statistically significant elevation in complication rates compared to the control cohort (47.4% vs. 20.4% for urinary tract infections, *p* < 0.001; 18.4% vs. 4.3% for pulmonary infections, *p* < 0.001), with infectious complications emerging as the most prominent manifestations. The delirium cohort demonstrated substantially prolonged critical care utilization (median ICU duration: 17.8 days vs. 0 days, *p* < 0.001) and increased total hospitalization requirements (median hospital stay: 13.0 days vs. 10.0 days, *p* < 0.001), reflecting greater healthcare resource consumption.

Univariate analysis (Table 2) identified multiple significant predictors of POD, including advanced age (> 82 years; OR = 3.34), prolonged preoperative waiting time (> 90 h; OR = 3.57), frailty (OR = 6.10), comorbidities (hypertension, respiratory diseases, stroke history), malnutrition (OR = 12.68), and infectious complications (urinary tract infections: OR = 3.52; pulmonary infections: OR = 5.00). Notably, nutritional indices (MNA scale), ASA classification and CCI demonstrated statistical significance in univariate models (all *p* < 0.05) but were excluded from the multivariate model. This exclusion likely reflects either collinearity with retained variables (e.g., frailty and age) or their potential role as secondary outcomes rather than independent etiological factors. Multivariable logistic regression analysis (see Table 3) identified four independent predictors of POD: frailty (OR = 2.62, *P* = 0.014), preoperative waiting time > 90 h (OR = 2.59, *P* = 0.007), postoperative infection (OR = 2.41, *P* = 0.013), and age > 82 years (OR = 2.44, *P* = 0.008). Collectively, these findings suggest that frailty, protracted preoperative waiting time, and postoperative infection are the primary factors associated with POD in this cohort. This provides an evidence-based foundation for refining perioperative assessment and guiding future interventional research.

**Table 2:** Binary logistic regression models for variables associated with the risk of POD.

Variable	OR 95% CI	*p* value
Age > 82 years**	3.34 (1.89 ~ 5.89)	< 0.001
Male	0.71 (0.37 ~ 1.33)	0.282
BMI, kg/m^2^	0.95 (0.89 ~ 1.01)	0.111
Active smoking	0.70 (0.18 ~ 2.66)	0.599
Alcohol abuse	0.42 (0.05 ~ 3.65)	0.430
Hypertension*	1.94 (1.03 ~ 3.63)	0.039
Heart disease	1.41 (0.81 ~ 2.47)	0.223
Diabetes mellitus	0.81 (0.45 ~ 1.48)	0.499
Kidney disease	1.07 (0.35 ~ 3.25)	0.904
Respiratory diseases*	2.56 (1.25 ~ 5.27)	0.011
History of stroke*	1.95 (1.08 ~ 3.5)	0.026
Mental disease	6.62 (0.68 ~ 64.69)	0.104
CCI**	1.27 (1.13 ~ 1.43)	< 0.001
MNA scale		
At risk vs. normal nutrition*	2.42 (1.1 ~ 5.32)	0.028
Malnourished vs. normal nutrition**	12.68 (4.09 ~ 39.37)	< 0.001
Frailty**	6.10 (3.28 ~ 11.34)	< 0.001
Type of fracture		
Intertrochanteric vs. Femoral neck	1.53 (0.88 ~ 2.67)	0.135
Subtrochanteric vs. Femoral neck	0.89 (0.09 ~ 8.8)	0.917
Side right vs. left	0.92 (0.53 ~ 1.6)	0.779
Multiple fractures	1.02 (0.45 ~ 2.28)	0.967
Contralateral hip fracture	1.54 (0.63 ~ 3.78)	0.346
Internal fixation vs. Arthroplasty	1.31 (0.76 ~ 2.27)	0.337
Spinal anesthesia vs. General anesthesia	0.78 (0.44 ~ 1.41)	0.420
Total albumin*, g/L	0.92 (0.87 ~ 0.97)	0.002
Albumin**, g/L	0.86 (0.8 ~ 0.93)	< 0.001
Absolute lymphocyte count, × 10⁹/L	0.81 (0.47 ~ 1.39)	0.436
PNI	0.86 (0.8 ~ 0.93)	< 0.001
Preoperative waiting time > 90 h**	3.57 (1.98 ~ 6.43)	< 0.001
ASA classification		
Class III vs. Class II*	11.53 (1.53 ~ 87.19)	0.018
Class IV vs. Class II**	32.00 (3.62 ~ 282.7)	0.002
Operation time, hours	0.79 (0.54 ~ 1.16)	0.235
Estimated blood loss, mL	1.00 (0.99 ~ 1)	0.194
Postoperative complications		
Venous Thromboembolism	1.07 (0.6 ~ 1.9)	0.819
Urinary tract infections**	3.52 (1.95 ~ 6.35)	< 0.001
Pulmonary infection*	5.00 (1.93 ~ 12.98)	0.001
Postoperative infection**	3.96(2.21 ~ 7.08)	< 0.001
Myocardial infarction	8.94 (0.98 ~ 81.44)	0.052
Stroke	2.69 (0.79 ~ 9.11)	0.112
Gastrointestinal bleeding	2.15 (0.13 ~ 34.79)	0.591
Hb on Day 1 post-operation, g/L	0.98 (0.96 ~ 1)	0.082

POD, postoperative delirium; BMI, Body mass index; CCI, Charlson comorbidity index; MNA scale, Mini nutritional assessment scale, "Nutritional status was assessed using the Mini Nutritional Assessment (MNA). Participants were classified into three groups based on MNA scores: malnourished (< 17), at risk of malnutrition (17–23.5), and normal nutritional status (≥ 24)."; PNI, Prognostic Nutritional Index; ASA, American society of anesthesiologists; ICU, intensive care unit.

*p value < 0.05 is statistically significant, **p value < 0.001 is highly significant.

**Table 3:** Multivariable logistic regression analysis for POD.

Variable	B		SE	Wald	*p* value	OR	95% CI
Constant	-4.140		1.126	13.521	< 0.001	0.02	
Age > 82 years**	0.891		0.338	6.947	0.008	2.44	1.26 to 4.73
Frailty*	0.961		0.390	6.070	0.014	2.62	1.217 to 5.62
MNA scale							
At risk vs. Normal	0.210		0.485	0.188	0.664	1.23	0.48 to 3.19
Malnourished vs. Normal	1.235		0.678	3.320	0.068	3.44	0.91 to 12.98
CCI score	0.054		0.075	0.508	0.476	1.06	0.91 to 1.22
Preoperative waiting time > 90 h**	0.952		0.350	7.403	0.007	2.59	1.305 to 5.15
ASA grade							
Class III vs. Class II	1.143		1.089	1.103	0.294	3.14	0.37 to 26.51
Class IV vs. Class II	1.596		1.208	1.747	0.186	4.93	0.46 to 52.61
Postoperative infection*	0.880		0.353	6.207	0.013	2.41	1.21 to 4.82

POD, postoperative delirium; B, regression coefficient; SE, standard error; OR, odds ratio; CI, confidence interval. CCI: Charlson comorbidity index; MNA scale, Mini nutritional assessment scale, “Nutritional status was assessed using the Mini Nutritional Assessment (MNA). Participants were classified into three groups based on MNA scores: malnourished (< 17), at risk of malnutrition (17–23.5), and normal nutritional status (≥ 24).”

The adjusted model included the following covariates: Age > 82 years, Preoperative waiting time > 90 h, Frailty, MNA Scale (At Risk, Malnourished), CCI Score, ASA Grade (III, IV), and Postoperative infection.

*p value < 0.05 is statistically significant, **p value < 0.001 is highly significant.

## Discussion

This study identifies frailty, a preoperative waiting time exceeding 90 h, and postoperative infection as independent and significant risk factors for POD in older hip fracture patients, even after adjustment for advanced age (> 82 years). Our findings regarding the association between frailty and delirium are consistent with previous observations across surgical cohorts^3,25,26^, and the adverse impact of delayed surgery has likewise been reported in earlier work^27,28^. The present analysis adds specificity by demonstrating that frailty remains an independent predictor even after adjustment for age and surgical timing, by identifying a data-driven threshold of 90 h for prolonged waiting time, and by confirming postoperative infection as an additional independent factor. The establishment of this threshold may facilitate the elucidation of the inconsistencies observed in previous studies that evaluated the clinical relevance of waiting time^28,29^. The correlation with postoperative infection underscores the potential contribution of inflammatory processes to the risk of delirium. Taken together, these findings offer clinically relevant information for risk stratification and perioperative decision-making, while acknowledging that the observational design precludes conclusions about causality or the effectiveness of specific interventions.

Our study demonstrates that preoperative frailty is a significant and independent predictor of POD in older patients with hip fractures (OR = 2.65), a finding that is consistent with and adds supportive evidence to the existing body of research^25,30,31^. The association between frailty and adverse postoperative outcomes, including delirium, has been repeatedly demonstrated in diverse surgical and hospitalized older populations. For instance, Leung et al. identified frailty as an independent risk factor for POD in non-cardiac surgery patients^26^, while a meta-analysis of 15 cohorts (*n* = 3,250) reported a frailty prevalence of 27.1% and a POD incidence of 15.8%, demonstrating an elevated risk associated with frailty (pooled OR = 3.23)^32^. The findings of this study are further supported by cohort-specific evidence from geriatric trauma patients studied by Esmaeeli et al.^33^ (*n* = 556, OR = 1.33) and from the total hip arthroplasty cohort reported by Chen et al.^34^ (*n* = 383, OR = 3.31), both of which also identified frailty as an independent predictor. By providing data from a well-characterized older hip fracture cohort, the results of this study add to and extend the accumulating evidence on the central role of frailty in delirium vulnerability.

The present analysis contributes to a more refined understanding of POD risk in hip fracture patients. Our findings show that frailty remains a robust and independent predictor even after comprehensive adjustment for factors such as advanced age and prolonged preoperative waiting time. This observation supports the view of frailty as a distinct state of physiological vulnerability, characterized by dysregulated inflammatory and stress-response pathways that may underlie key pathogenic mechanisms^17–20^. The integration of a standardized frailty assessment within the preoperative evaluation process has the potential to facilitate the early identification of high-risk patients who could benefit from targeted perioperative strategies, including geriatric co-management or prehabilitation. It is evident from the aforementioned findings that assessments and interventions targeting the physiological pathways central to frailty are of paramount importance when seeking to enhance perioperative care^21^.

The multivariate analysis indicates that advanced age (> 82 years) remains an independent predictor of POD (OR = 2.44). This observation supports the view that the influence of age persists even after adjusting for clinically relevant factors such as frailty and preoperative waiting time. The study emphasizes the significance of age as a reflection of cumulative biological susceptibility. The association aligns well with the existing literature describing the relationship between older age and delirium, a link often attributed to age-related reductions in physiological reserve, compromised neurovascular function, and a greater comorbidity burden^35–37^.

The critical refinement offered by our study lies in the identification of a specific threshold (> 82 years) within a hip fracture cohort and its demonstration as an independent factor after adjustment for other modifiable factors. This indicates that in the very old, biological aging processes may create a substrate of heightened neuroinflammatory susceptibility and reduced physiological resilience^17,19,38,39^. Consequently, advanced age may serve as a crucial, independent risk indicator, warranting particular attention in the perioperative risk stratification of geriatric hip fracture patients.

Similarly, prolonged preoperative waiting time (> 90 h) was identified as a potent and modifiable risk factor for POD (adjusted OR = 2.59). As is well-established, there is a detrimental association between surgical delay and postoperative complications^27–29^. However, the present analysis offers greater precision and refinement to this understanding. The present study proposes a high-risk threshold of 90 h, independent of frailty and chronological age, thereby providing healthcare systems with a pragmatic, evidence-based operational target. This delineated threshold also offers a plausible explanation for the variability reported in previous studies^40–42^, suggesting that the escalation in delirium risk may occur predominantly beyond this critical temporal tipping point. These findings emphasize the necessity of implementing expedited surgical pathways with a view to mitigating preventable risk in this vulnerable population.

The findings of this study demonstrate that the risk associated with waiting is not merely a function of elapsed time, but rather appears to escalate once a biological tipping point is exceeded, potentially through compounded pain, stress, inflammation, and metabolic derangement^43–45^. Consequently, in conjunction with system-level endeavours to minimise delays, proactive prehabilitation strategies that mitigate these physiological stressors may hold particular significance for patients who encounter unavoidable waiting periods prior to surgery^44,45^.

Furthermore, this prospective study confirms postoperative infection as an independent risk factor for POD in older hip fracture patients. This finding aligns with previous research^46^, and the prospective design offers a clearer temporal sequence between postoperative infection and subsequent delirium compared with retrospective analyses. In these vulnerable patients, infection may function as a systemic stressor rather than a localized event, potentially contributing to neuropsychiatric manifestations through inflammatory mediator cascades and metabolic disturbances. While acknowledging the multifactorial origins of delirium, the prospectively validated independent association with infection underscores its clinical significance. Incorporating rigorous infection-control measures—such as vigilant monitoring for early signs, strict adherence to aseptic technique, and minimizing invasive device use—into broader delirium-prevention protocols may help reduce the risk of POD and support better long-term outcomes for patients^47^.

In contrast, neither the type of anaesthesia nor the surgical procedure showed a significant association with the incidence of postoperative delirium (POD), which is consistent with recent meta-analyses^16^. This suggests that these perioperative management choices are not the main determinants of delirium risk in this population. Instead, our findings reinforce the idea that intrinsic patient vulnerability, as measured here by frailty, plays a more pivotal role. This highlights the importance of prioritizing comprehensive geriatric assessment over procedure-centric approaches.

The present study demonstrates that POD is associated with important clinical sequelae, including a markedly higher incidence of myocardial infarction (5.3% vs. 0.6%) and a prolonged ICU length of stay, consistent with previous reports of increased resource utilization^48,49^. The potential for a pathophysiological relationship between delirium and cardiac events may be attributable to shared mechanisms, such as systemic inflammation^19^. The higher incidence of POD in our cohort compared with that reported by Hernandez et al. in a general surgical population (31.9% vs. 16.4%)^50^, highlights the substantial baseline vulnerability characteristic of geriatric patients with hip fractures. It is important to note that our model identified frailty, rather than the more conventional ASA classification, as a primary predictor. This finding suggests that conventional perioperative risk tools may inadequately capture the dimension of physiological reserve that is most relevant for older adults. The observed discrepancy from prior findings^16^ may be attributed to the distinctively high vulnerability inherent to the hip fracture population, which is not fully captured by conventional perioperative risk assessments.

This study has several limitations. The single-center design and the absence of inflammatory biomarker assessment (e.g., IL-6, CRP) may limit the generalizability of our findings. While clinically representative, the inclusion of patients with preexisting dementia in our frailty assessment may introduce selection bias. Furthermore, the exclusion of patients unable to consent or complete baseline tests may limit the applicability of our findings to the most vulnerable patients. Our focus on new-onset delirium precluded analysis of preoperative delirium as a risk factor. Additionally, the dichotomization of continuous variables (age and preoperative waiting time) may introduce bias and result in a loss of statistical information, although it was performed to identify clinically actionable thresholds. Most importantly, the observational nature of this study, while identifying significant associations, cannot establish causality between identified risk factors and POD due to potential residual confounding. Finally, this study focused on in-hospital outcomes; long-term survival was not assessed.

Consequently, future research should prioritize multicentre studies to validate the identified risk model. This would address the above-noted limitations and confirm the robustness of our core findings. Of particular significance is the potentially modifiable nature of the key predictors identified in this study: frailty and prolonged preoperative waiting time. These observations provide a strong rationale for interventional research. Randomized controlled trials evaluating targeted prehabilitation programs to mitigate frailty, alongside clinical pathway interventions aimed at reducing time-to-surgery towards or below the observed 90-hour threshold, are promising next steps in determining whether modifying these factors can causally reduce POD, and in supporting their translation into effective clinical practice.

## Conclusion

This study identifies frailty, prolonged preoperative waiting time (> 90 h), and postoperative infection as important and potentially modifiable predictors of POD in older hip fracture patients, while advanced age (> 82 years) as a non-modifiable risk indicator. These findings highlight the potential value of incorporating routine frailty assessment, streamlined surgical pathways, and strengthened infection control into perioperative management strategies aimed at reducing delirium risk. By quantifying the associated healthcare burden, including prolonged ICU stays, the results also underscore the potential economic implications of targeted preventive approaches. Further randomized controlled trials are recommended to validate this multi-component intervention strategy.

## Data Availability

The corresponding author can provide the raw data from this study upon reasonable request.
